# Insecticide Susceptibility Status of *Anopheles* and *Aedes* Mosquitoes in Malaria and Dengue Endemic Areas, Thai–Myanmar Border

**DOI:** 10.3390/insects13111035

**Published:** 2022-11-09

**Authors:** Kanchon Pusawang, Jetsumon Sattabongkot, Jassada Saingamsook, Daibin Zhong, Guiyun Yan, Pradya Somboon, Somsakul Pop Wongpalee, Liwang Cui, Atiporn Saeung, Patchara Sriwichai

**Affiliations:** 1 Center of Insect Vector Study, Department of Parasitology, Faculty of Medicine, Chiang Mai University, Chiang Mai 50200, Thailand; 2 Mahidol Vivax Research Center, Faculty of Tropical Medicine, Mahidol University, Bangkok 10400, Thailand; 3 Department of Population Health and Disease Prevention, University of California, Irvine, CA 92697, USA; 4 Department of Microbiology, Faculty of Medicine, Chiang Mai University, Chiang Mai 50200, Thailand; 5 Division of Infectious Diseases, Department of Internal Medicine, Morsani College of Medicine, University of South Florida, Tampa, FL 33612, USA; 6 Department of Medical Entomology, Faculty of Tropical Medicine, Mahidol University, Bangkok 10400, Thailand

**Keywords:** *An. minimus*, *An. maculatus*, *Ae. aegypti*, bendiocarb, deltamethrin, *kdr* mutations, pyrethroids, dengue, malaria

## Abstract

**Simple Summary:**

This study determined the insecticide susceptibility of malaria and dengue vectors in a co-existing hotspot area, the Thai–Myanmar border. The insecticide resistance for the Pyrethroids group and the genetic resistance were revealed in *Aedes aegypti*, and the emergence of the *Anopheles* malaria vectors resistance was detected. Routine malaria and dengue vector control programmes, such as fogging implementation in the hotspot villages to induce higher mosquito vector resistance available in peri-domestic sites, are questionable. This occurrence is the informative data for the routine monitoring of vector controls to avoid the emergence of insecticide resistance among mosquitoes.

**Abstract:**

The occurrence and spread of insecticide resistance has had a negative effect on the efficacy of insecticide–based tools and is distributed worldwide, including the Greater Mekong Subregion (GMS). This study aims to determine the insecticide susceptibility of malaria and dengue vectors in malaria and dengue hotspots on the Thai–Myanmar border. Mosquito larvae and pupae were obtained from water sources from December 2019 to April 2020 in Tha Song Yang District, Tak province, western Thailand. WHO bioassay susceptibility tests were conducted with three classes of insecticides to evaluate the knockdown and mortality rates of *Anopheles* and *Aedes aegypti* female adults. V1016G and F1534C *kdr* mutations in the voltage-gated sodium channel of *Ae. aegypti* were identified using a multiplex PCR. A total of 5764 female mosquitoes were bioassayed in this study, including *Anopheles* spp. (92.63%) and F1 *Ae. aegypti* (7.37%). After 24 h of observation, *An. minimus* s.l. (*n* = 3885) and *An. maculatus* s.l. (*n* = 1138) in Suan Oi (SO) and Tala Oka (TO) were susceptible to pyrethroids, organophosphates and carbamates (except bendiocarb) with 98–100% mortality (MR). Resistance to bendiocarb was detected with a mortality rate of 88.80%, 88.77%, and 89.92% for *An. minimus* s.l. (*n* = 125, 125) and *An. maculatus* s.l. (*n* = 66), respectively. The first generation of *Ae*. *aegypti* adult females were suspected of resistance to deltamethrin (*n* = 225, MR = 96.89%) and confirmed resistance to permethrin (*n* = 200, MR = 20.00%). V1016G and F1534C mutations were detected in three genotypes, heterozygote and homozygote forms. The correlation between the *kdr* alleles and deltamethrin resistance was significant. In conclusion, bendiocarb resistance was found in primary malaria vectors, *An. minimus* s.l. and *An. maculatus* s.l. F1 *Ae. aegypti* population was pyrethroids-resistant, associated with *kdr* alleles. Therefore, molecular analysis should be conducted to gain insights into the mechanism of insecticide resistance. Routine malaria vector control programmes, such as fogging implementation in hotspot villages to induce *Aedes* resistance available in peri–domestic sites, are questionable.

## 1. Introduction

Malaria control programmes currently rely on insecticide-treated bed nets (ITNs), indoor residual spraying (IRS), and antimalarial drugs, all of which helped to reduce malaria cases worldwide from 238 million in 2000 to 229 million in 2019 [1,2], although a subsequent upsurge in cases has added new urgency to malaria prevention campaigns. Compared to Africa, the use of ITNs in the Greater Mekong Subregion (GMS) is relatively less extensive but has nonetheless given impressive malaria control outcomes in many studies [3,4,5,6]. Several markable synthetic insecticides have been applied to control mosquito-borne diseases over decades, including organochlorines (DDT), organophosphates (temephos), carbamates (propoxur) and pyrethroids (permethrin, deltamethrin) [7]. Due to their strong lethal effects and low toxicity to humans [8,9], pyrethroids are currently the only approved insecticides used for ITNs [10]. Unfortunately, the rapid spread of insecticide resistance poses a serious challenge to vector control programmes worldwide, as shown in the slow decline in morbidity since 2015 [1]. High coverage of vector control interventions and agricultural purposes cause mosquitoes to develop resistance to these insecticides [11].

There are two major insecticide resistance mechanisms in insects: metabolic resistance and target–site resistance [12]. Metabolic resistance refers to the increase in insecticide metabolism through the overproduction of cytochrome P450 [13,14], esterase [15,16], and glutathione S-Transferases [17]. Target site insensitivity is inferred when mutation of the insecticide target site occurs and causes limiting of the insecticidal effects [18]. These modifications usually include the voltage-sensitive sodium channel (*Vssc*) mutation, commonly known as knockdown resistance (*kdr*) in DDT and pyrethroid [19], insensitivity of acetylcholinesterase (AChE1), which is a target of organophosphate and carbamate [20], and GABA receptor mutation responsible for cyclodiene, fipronil and pyrethroid insensitivity [21].

In Thailand, malaria and dengue fever are the dominant vector–borne diseases. A total of 3,051 malaria cases and 9,494 dengue cases were reported in 2021 [22,23]. The number of dengue cases was drastically reduced from 2020 (72,519 cases), which might be because of the COVID–19 pandemic. Resistance to deltamethrin and/or permethrin was observed in populations of *Ae*. *aegypti* throughout Thailand [23,24,25,26,27,28,29,30]. Primary *kdr* mutations have been identified and verified to be associated with pyrethroid resistance in previous studies, which are commonly base substitutions V1016G, F1534C, and S989P (often occurs with V1016G) [25,28,31,32]. The absence of *kdr* mutation in prominent malaria mosquitoes, i.e., *An. minimus* and *An. maculatus,* was found and metabolic resistance is a causative mechanism [33,34]. An increase in enzyme expression involved in metabolic resistance was found with a high activity of P450s (*CYP9J32*, *CYP9J24*, *CYP9J26*, and *CYP9J28*), and carboxylesterase genes (*CCEae3a*, *CCEae6a*) found in resistant *Ae. aegypti* samples [35,36]. In the *An. minimus* laboratory strain, the mRNA expression level of *CYP6AA3* and *CYP6P7* in resistant individuals was greater and correlated with increased resistance to pyrethroids [37,38].

This study assesses the insecticide susceptibility status in *Anopheles* spp. and *Ae. aegypti* mosquitoes to WHO adulticides along the Thai–Myanmar border. To better understand the resistance mechanism in *Ae. aegypti*, particularly in malaria and dengue co-existing endemic areas, the prevalence of *kdr* alleles and their correlation with observed phenotypes were also noted.

## 2. Materials and Methods

### 2.1. Study Sites and Mosquito Collections

**Figure 1:**
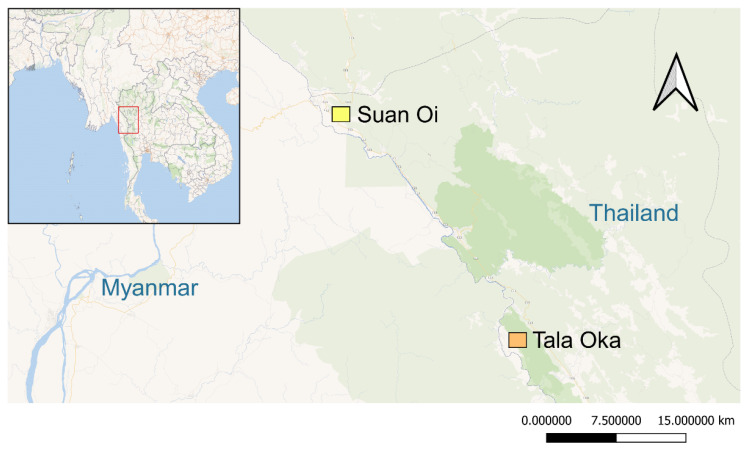
Map of Tak province (red frame) and collection sites of *Anopheles* spp. and *Ae. aegypti*, Suan Oi (SO); Tala Oka (TO). These two villages are located along the Thai–Myanmar border.

The study sites comprised two villages—Suan Oi (SO, 17°56′ N, 97°91′ E) and Tala Oka (TO, 17°33′ N, 98°10′ E) in Tha Song Yang District, Tak Province, western Thailand, on the Thai–Myanmar border, a malaria hotspot area divided by the Moei River (Figure 1). At the time of our work, there were 596 and 1,218 residents in SO and TO, respectively. About 50 water sources were found in the two villages and located near households. Records provided by the Bureau of Vector-Borne Diseases showed malaria incidences of 441 cases in 2021, most of which were caused by *P. vivax* [22]. In addition, 42 dengue cases were reported in Tha Song Yang District [23]. Malaria incidence occurs all year round and peaks after the wet season. The primary malaria vectors in this area are *An. minimus*, *An. maculatus* and *An. dirus* [6]. According to Thailand’s vector control policy, indoor residual spraying is conducted twice a year. Pyrethroid-treated bed nets (deltamethrin and permethrin) were distributed to local residents for malaria control. The larvicide temephos, sand granules, and fogging with deltamethrin were used to control dengue transmission.

### 2.2. Mosquito Collection

Entomological surveys were conducted from December 2019 to April 2020. *Aedes* larvae and pupae were obtained from domestic water containers, and *Anopheles* larvae and pupae were sampled from 50 stream sites by the dipping method. All larvae were kept alive in 400 mL plastic bottles and taken to an insectary for rearing in the laboratory. The species were identified based on morphological characters [39].

### 2.3. Mosquito Rearing

Mosquito rearing procedures followed the detailed techniques described by Choochote and Saeung [40]. Larvae and pupae were transferred to plastic trays (25 cm × 35 cm × 6 cm) containing 1 L of natural streaming water near the village. About 80 larvae were placed in each rearing tray. Tetramin^TM^ fish food was fed to the mosquito larvae daily. The trays were refilled with water when needed. Pupae were transferred into adult emergence cages (30 cm × 30 cm × 30 cm), and 5–7-day-old adult female mosquitoes were used for species identification and further analysis. To increase the sample size of *Ae. aegypti*, the emerged female *Ae. aegypti* were allowed to feed on blood through an artificial membrane feeder [40]. Subsequently, fully gravid mosquitoes were placed inside a plastic cup containing water for oviposition.

### 2.4. Insecticide Susceptibility Test

Wild-type *Anopheles* spp. and the first generation of *Ae. aegypti* were tested in this study. Bioassays were performed on adult mosquitoes using the standard WHO susceptibility bioassay test [41]. Insecticide–impregnated papers and controls were supplied by the Vector Control Research Unit, University of Sains Malaysia. Briefly, 20–25 female mosquitoes were transferred to a holding tube in an upright position for one hour. The dead mosquitoes were removed. After that, the remaining mosquitoes were blown through the opened slit to exposure tubes. They were kept in the test tube for one hour. The number of knocked-down mosquitoes was recorded every five minutes until 60 min. After one hour of exposure, mosquitoes were transferred gently into holding tubes. A 10% sugar solution was provided as food for adults on top of the net screen. Tubes containing mosquitoes were kept in the laboratory at 25 ± 2 °C, 70–80% relative humidity for 24 h. Mortality was recorded after a 24 h observation period. All tested materials were preserved in absolute ethanol.

### 2.5. DNA Extraction and kdr Detection in Aedes aegypti

Detection of *kdr* alleles was performed only for *Ae*. *aegypti* because most *Anopheles* mosquitoes died after being bioassayed. Genomic DNA was extracted from the whole body of resistant (survivor) and susceptible (dead) mosquitoes using PureLink™ Genomic DNA Mini Kit (Invitrogen, Carlsbad, CA, USA), according to the manufacturer’s instructions. DNA quantity was determined using a Nanodrop 2000 (Thermo Scientific, Delaware, ME, USA). Genotyping of the *kdr* alleles was conducted using the multiplex PCR developed by Saingamsook et al. [28]. The V1016G and F1534C mutations of the voltage-gated sodium channel (*Vgsc*) were detected using seven primers (0.5 µM Gly1016f, 0.25-µM Val1016r, 0.5-µM Gly1016r, 0.25-µM c1534–f, 0.25-µM c1534-r, 0.1-µM Ae1534F-r, and 0.5-µM Ae1534C-f). Each PCR reaction was conducted with 10-µL volumes containing 0.4-U Taq DNA polymerase, 1-µL 10X buffer, 0.2-mM of each dNTP, each primer at a concentration as described, and 1-µL DNA template. The PCR programme comprised initial denaturation at 95 °C for 2 min, 35 cycles of denaturation at 95 °C for 30 s, annealing at 55 °C for 30 s, and extension at 72 °C for 30 s, with a final extension at 72 °C for 2 min. The amplified products were electrophoresed on 2% agarose gel and stained using ethidium bromide.

### 2.6. Data Analysis

The results were interpreted according to WHO guidelines 2016 [41]. Mosquitoes were considered resistant (R) if the mortality was less than 90%, suspected resistant if the mortality rate was between 90–97%, and susceptible (S) if the mortality rate was greater than 98%. Median knockdown time (KDT50) was determined through the Probit analysis using IBM SPSS statistics 24 (IBM Corp., Armonk, NY, USA). Fisher’s exact test was used to determine the association between resistant and susceptible mosquitoes and their *kdr* genotypes using GraphPad Prism version 8.1 (GraphPad Software, San Diego, CA, USA).

## 3. Results

### 3.1. Bioassays

**Figure 2:**
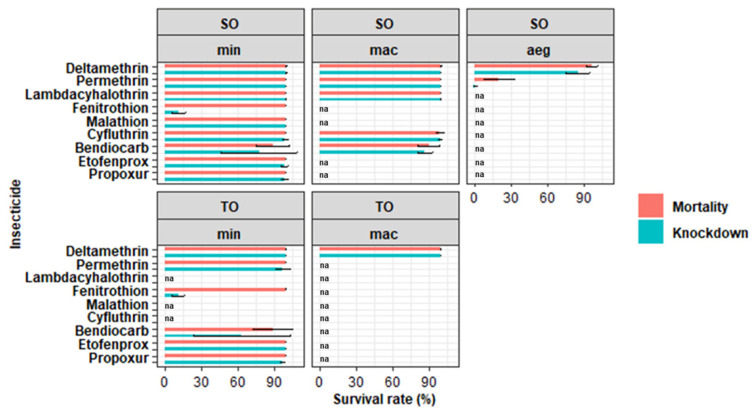
Mortality and knockdown rate assessed following the WHO guidelines for insecticide monitoring in *An. minimus* s.l. (min), *An. maculatus* s.l. (mac), and *Ae. aegypti* (aeg) from Suan Oi (SO) and Tala Oka (TO) villages. Error bars represent standard error; na represents no data values.

**Figure 3:**
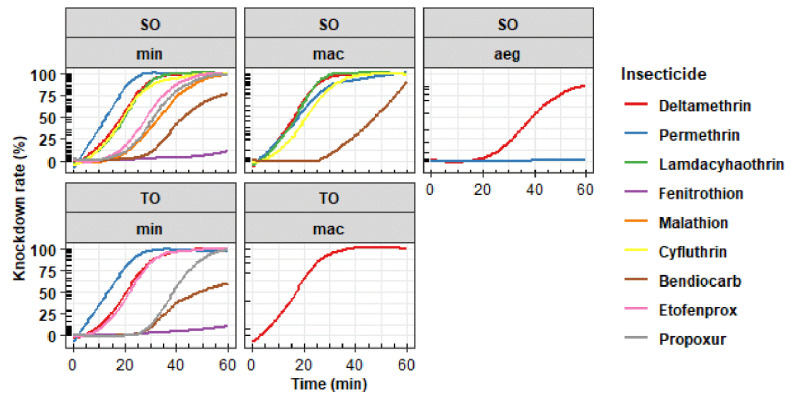
Knockdown rate of *An. minimus* s.l. (min), *An. maculatus* s.l. (mac) and *Ae. aegypti* (aeg) from Suan Oi (SO) and Tala Oka (TO) villages against different classes of insecticide.

A total of 5764 female mosquitoes were obtained from two villages—Suan Oi (SO) and Tala Oka (TO) in Tha Song Yang District—and tested in this study. Among these, 5339 (92.63%) were wild-type *Anopheles* spp. and 425 (7.37%) were F1 *Ae. aegypti* (from about 200 parents). The mortality and knockdown (KD) time for *An. minimus* s.l., *An*. *maculatus* s.l., and *Ae*. *aegypti* in Suan Oi (SO) are shown in Table 1, and the corresponding data for *An*. *minimus* s.l. and *An*. *maculatus* s.l. in Tala Oka (TO) are shown in Table 2. Figure 2 shows the mortality and knockdown rate in *An. minimus* s.l. (min), *An. maculatus* s.l. (mac) and *Ae. aegypti* (aeg) from both study sites. *Anopheles minimus* s.l. and *An. maculatus* s.l. in both sites were susceptible to most of the insecticides, with the mortality rate ranging between 98% and 100%, except for bendiocarb. Resistance to 0.1% bendiocarb was found in *An. minimus* s.l. at both sites and *An. maculatus* s.l. in SO, and the mortality rates were 88.80%, 88.77%, and 89.92%, respectively. The kinetic graphs of knockdown rates are presented in Figure 3. In F1 *Ae. aegypti*, suspected resistance to deltamethrin (mortality 96.89%) and resistance to permethrin (mortality 20%) was found. The KDT50 values for deltamethrin and permethrin were 41.09 and 144.33 min, respectively.

### 3.2. Prevalence of kdr Mutations in Ae. aegypti

*Kdr* genotypes and frequency of G and C alleles of tested *Ae. aegypti* (F1) (*n* = 97) are shown in Table 3. The *kdr* genotypes comprised the homozygous V1016/C1534 (VV/CC) (68/97), the heterozygous V1016G/F1534C (VG/FC) (23/97), and the homozygous G1016/F1534 (GG/FF) (6/97) mutations. The mosquitoes were deltamethrin-resistant and harboured a significantly higher frequency of the G mutant allele (0.714) compared with the susceptible mosquitoes (0.167), which harboured high frequency of the C allele (0.833) (*p* < 0.001). The groups of mosquitoes that were resistant and susceptible to permethrin did not show significant differences in the frequency of G or C alleles (*p* > 0.5).

Through Fisher’s exact test, an association between *kdr* alleles and pyrethroids (deltamethrin/permethrin) resistance was calculated. It was found that the probability of being resistant to deltamethrin of the mosquitoes harbouring the G allele (Odd ratio) was 12.50 times and 0.08 times for the C allele (*p* = 0.0001), but there was no association between mutant alleles and permethrin resistance (G allele: *p* = 1.588, F allele: *p* = 0.583) (Table 4).

## 4. Discussion

In this study, the susceptibility of *An. minimus* s.l., *An. maculatus* s.l. and *Ae. aegypti* against different pyrethroids, organophosphates and carbamates was determined according to WHO guidelines from 2019–2020. The Thai–Myanmar border (TMB) was noted as a hotspot for malaria and other important vector–borne diseases [6]. Indeed, vector control interventions such as long-lasting insecticide-treated nets (LLINs) and indoor residual spraying (IRS) have been widely used. Our findings revealed the spread of insecticide resistance in the local mosquito population. Bendiocarb resistance was found in *An. minimus* s.l. and *An. maculatus* s.l. In addition, pyrethroid resistance and *kdr* alleles (1016G, 1534C) were detected in *Ae. aegypti.*

In common with findings from previous studies [33,42], *An. minimus* s.l. was the most abundant species in SO and TO villages, followed by *An. maculatus* s.l. Both species contain *P. vivax* CS proteins, representing the malaria vector competency, and acted as important vectors in this area. No pyrethroid resistance in *Anopheles* spp. was noticed in our observation, whereas suspected resistance to deltamethrin in *An. minimus* s.l. has been reported in TMB [33]. This might be because of the frequency of insecticide use for public health programmes and agriculture in each location. The resistance to bendiocarb in *An. minimus* s.l. and *An. maculatus* s.l. in these villages was discovered. It has been found in recent studies, especially in sub–Saharan Africa [43,44,45,46], where *An. gambiae* developed resistance to bendiocarb with the presence of G119S induced by the *ace–1*^R^ gene. Cytochrome P450s also confer metabolic resistance to bendiocarb [47]. *Anopheles minimus* s.l. in this study was susceptible to propoxur, which is in the same class as bendiocarb. Bendiocarb is an insecticide class named carbamate. It was formerly used as an alternative insecticide when the spread of pyrethroid resistance occurred in some regions of Africa [43]. According to LLIN campaigns, bendiocarb is not the most commonly used insecticide in Thailand, compared with pyrethroids, and the origin of bendiocarb resistance remains unknown.

Our study showed that pyrethroid resistance (deltamethrin and permethrin) with *kdr* alleles was observed in F1 *Ae*. *aegypti* originated from TMB. This is the first report of *kdr* mutation detection in *Ae*. *aegypti* in TMB. G1016 and C1534 *kdr* mutant alleles are widely distributed globally and associated with resistance to pyrethroids in numerous studies [25,27,32,48,49]. V1016G mutation was found to be associated with resistance to type I (permethrin) and type II (deltamethrin) pyrethroids [25,31], whereas mosquitoes harbouring F1534C mutation conferred type I pyrethroid resistance [50].

Our study showed that high G1016 allele frequency increased the likelihood of becoming resistant to deltamethrin, but this was decreased when the mosquitoes had a high C1534 allele frequency, showing that the G allele was associated with deltamethrin resistance. Similarly, Saudi Arabia [51] showed a higher survival advantage (Odd ratio) of GG/FF/PP(S989P) genotype *Ae*. *aegypti* compared with VV/CC/SS and VG/FC/SP genotypes, when exposed to 0.05% deltamethrin. However, the effect of 0.75% permethrin in our report was not obvious. This could be because of various concentrations of permethrin. A study in *Ae*. *aegypti* from Malaysia [52] indicated a survival advantage from the triple heterozygote (V1016G/F1534C/S989P) and homozygous mutant for the C1534 allele (V1016/C1534/S989) individuals compared with wild-type genotype against 0.25% permethrin. It is also essential to note that only seven dead mosquitoes were found and tested for multiplex PCR compared with 30 susceptible samples.

Usually, malaria transmission is confined to forest areas, while dengue outbreaks occur in urban and suburban areas. In our study villages, however, both diseases co-exist. Although no resistance was found in *Anopheles* vectors in this study, pyrethroids are used to control both diseases. Hence, the resistance of malaria vectors might develop faster than in areas with malaria alone. A previous study in Senegal [53] observed high pyrethroid resistance in *Ae*. *aegypti* in the central region, where malaria prevalence was high and increasing from the central to the southern regions [54]. For an effective control strategy, insecticide resistance of malaria vectors should be monitored continuously, and other alternative control methods in areas with resistance problems need to be incorporated.

Not only target site resistance is involved in the insecticide resistance of mosquitoes, but metabolic resistance is also the primary mechanism of resistance to pyrethroids. Biochemical assays suggested that metabolic resistance mechanisms might play an essential role in insecticide resistance in major malaria vectors in the Mekong region, including *An. minimus* s.l. and *An. dirus* s.s. [55]. To ensure the insecticide susceptibility of vectors, other approaches should be applied. For example, transcriptome analysis and whole genome analysis have been used to detect the variant between resistant and susceptible mosquitoes [56,57]. Even though pyrethroid resistance was found only in *Ae. aegypti* in this area, routine monitoring of both vectors needs to be conducted to prevent further emergence of insecticide resistance in mosquitoes.

## 5. Conclusions

This study demonstrated the spread of insecticide resistance in the natural mosquito population from malaria and dengue in co-existing areas. Bendiocarb resistance was found in both malaria vectors, *An. minimus* s.l. and *An. maculatus* s.l. Pyrethroids resistance and *kdr* alleles (1016G, 1534C) were detected in *Ae. aegypti.* Pyrethroids are locally used to control both diseases. Faster development of malaria vector resistance might be involved. This study provides informative data for the routine monitoring of both vector controls to avoid the further emergence of insecticide resistance among mosquitoes.

## Figures and Tables

**Table 1:** Summary results of WHO bioassay tested in *An. minimus* s.l., *An. maculatus* s.l., and *Ae. aegypti* in Suan Oi (SO).

Species	Insecticide	N ^1^	R ^2^	% Mortality	% Knockdown Rate	KDT50 ^3^ (min)	Status ^4^
*An*. *minimus* s.l.	0.05% Deltamethrin	1562	65	99.93 (99.80–100.07)	99.93 (99.80–100.07)	19.12 (14.45–23.55)	S
	0.75% Permethrin	96	4	100 (na)	100 (na)	13.72 (11.18–16.17)	S
	0.05% Lambdacyhalothrin	293	12	100 (na)	100 (na)	20.12 (18.16–20.06)	S
	1.0% Fenitrothion	195	8	100 (na)	11.25 (6.34–16.17)	105.47 (88.66–140.01)	S
	5.0% Malathion	50	2	100 (na)	100 (na)	34.10 (32.14–36.05)	S
	1.5% Cyfluthrin	75	3	100 (na)	98.67 (92.93–104.40)	21.47 (8.44–32.01)	S
	0.1% Bendiocarb	125	5	88.80 (71.81–105.79)	77.60 (38.87–116.33)	45.63 (43.36–48.14)	R
	0.5% Etofenprox	246	10	100 (na)	98.80 (96.87–100.73)	29.22 (28.18–30.26)	S
	0.1% Propoxur	444	18	100 (na)	98.89 (97.56–100.22)	31.72 (30.61–32.83)	S
*An*. *maculatus* s.l.	0.05% Deltamethrin	809	34	99.87 (99.61–100.13)	100 (na)	17.16 (7.80–25.23)	S
	0.75% Permethrin	100	4	100 (na)	100 (na)	19.77 (16.87–22.47)	S
	0.05% Lambdacyhalothrin	50	2	100 (na)	100 (na)	17.17 (16.36–17.97)	S
	1.5% Cyfluthrin	129	6	98.66 (95.24–102.09)	99.33 (97.62–101.05)	22.17 (17.48–26.48)	S
	0.1% Bendiocarb	66	3	89.92 (68.40–111.43)	86.50 (71.44–101.56)	46.95 (44.76–49.37)	R
*Ae*. *aegypti*	0.05% Deltamethrin	225	9	96.89 (93.19–100.58)	85.33 (77.65–93.02)	41.09 (39.87–42.33)	R*
	0.75% Permethrin	200	8	20 (9.43–30.57)	0.5 (–0.68–1.68)	144.33 (na)	R

^1^ Number of tested mosquitoes. ^2^ Number of replicates. ^3^ Time taken in minutes. ^4^ Status: S, susceptible (mortality 98–100%); R*, suspected resistance (mortality 90–97%); R, resistance (mortality < 90%). The values between brackets indicate the 95% confidence interval. Na: not available.

**Table 2:** Summary results of WHO bioassay tested in *An. minimus* s.l. and *An. maculatus* s.l. in Tala Oka (TO).

Species	Insecticide	N ^1^	R ^2^	% Mortality	% Knockdown Rate	KDT50 ^3^ (min)	Status ^4^
*An. minimus* s.l.	0.05% Deltamethrin	575	25	100 (na)	100 (na)	21.96 (18.96–24.83)	S
	0.75% Permethrin	100	4	100 (na)	97 (87.45–106.55)	15.44 (–14.67–28.78)	S
	1.0% Fenitrothion	100	4	100 (na)	11 (2.99–19.01)	104.69 (88.37–137.32)	S
	0.1% Bendiocarb	125	6	88.77 (71.88–105.66)	63.31 (21.18–105.44)	50.81 (47.60–54.95)	R
	0.5% Etofenprox	50	2	100 (na)	100 (na)	22.93 (19.59–26.14)	S
	0.1% Propoxur	99	4	100 (na)	96.96 (93.73–100.19)	39.56 (37.52–41.61)	S
*An. maculatus* s.l.	0.05% Deltamethrin	50	2	100 (na)	100 (na)	18.01 (16.59–19.40)	S

^1^ Number of tested mosquitoes. ^2^ Number of replicates. ^3^ Time taken in minutes. ^4^ Status: S, susceptible (mortality 98–100%); R, resistance (mortality < 90%). The values between brackets indicate the 95% confidence interval. na: not available.

**Table 3:** Genotype and allele frequencies of the V1016G, F1534C *kdr* mutations in F1 *Ae. aegypti* from Suan Oi village.

Insecticide	Status ^1^	Total PCR	*Kdr* Genotype			G Allele Frequency (95% CI)	C Allele Frequency (95% CI)
			VV/CC	VG/FC	GG/FF		
0.05% Deltamethrin	R	7	0	4	3	0.714 (0.454–0.883)	0.286 (0.117–0.546)
	S	30	22	6	2	0.167 (0.093–0.280)	0.833 (0.720–0.907)
0.75% Permethrin	R	30	22	7	1	0.150 (0.081–0.261)	0.850 (0.739–0.919)
	S	30	24	6	0	0.100 (0.047–0.201)	0.900 (0.799–0.953)

^1^ R (Resistant), S (Susceptible).

**Table 4:** Association between V1016G and F1534C alleles with resistance phenotype to deltamethrin and permethrin in adult *Ae. aegypti*.

Mutant Allele	Insecticide	Odd Ratio (95% CI)	Fisher’s Exact Test
1016G	0.05% Deltamethrin	12.500 (3.489–39.980)	0.0001 *
	0.75% Permethrin	1.588 (0.534–4.904)	1.588
1534C	0.05% Deltamethrin	0.080 (0.025–0.287)	0.0001 *
	0.75% Permethrin	0.630 (0.204–1.873)	0.583

* Significance difference.

## Data Availability

The data presented in this study are available in the article.
